# The impact of users' trust on intention to use the mobile medical platform: Evidence from China

**DOI:** 10.3389/fpubh.2023.1076367

**Published:** 2023-03-21

**Authors:** Jinjiang He

**Affiliations:** School of Business and Administration, Zhejiang University of Finance and Economics, Hangzhou, China

**Keywords:** mobile medical platform, use intention, innovation acceptance, technical risk concerns, trust

## Abstract

**Introduction:**

The mobile medical platform effectively complements offline medical services as it can provide patients with broader and more convenient medical services, effectively solving the shortage of medical resources in the public health system. Although the public interest in healthcare service platforms continues to rise, the market data shows that the adoption and acceptance have not reached a high level. How to increase the utilization rate of the mobile medical platform to relieve medical pressure has become an urgent issue to be discussed. Based on the framework of “trust-intention” this research introduces innovation acceptance and technical risk concerns as the two moderating variables to attempt to build a research model of users' intention to use the mobile medical platform. The analysis illustrated that users' trust in the mobile medical platform would positively affect their use intention. The researchers further explored the moderating role of innovation acceptance and technical risk concerns.

**Methods:**

Use questionnaire to collect data in China, then use the OLS least square method for the regression test.

**Results:**

The results showed that users with high personal innovation acceptance would positively promote the relationship between trust and use intention. In contrast, users who are more concerned about the risks of innovative technologies will weaken the relationship between trust and use intention.

**Discussion:**

The findings theoretically extend the academic research of use intention to the specific context of the mobile medical platform and enrich the research framework of “trust-intention”.

## 1. Introduction

The global spread of COVID-19 threatens human health and challenges the current level of medical services. The sheer number of people infected during the same period led to hospitals being the most severely infected areas. Thus, many countries and regions have introduced home isolation policies to alleviate the tremendous pressure of the shortage of medical resources, which has caused difficulties for people to access medical care. The mobile medical platform has received unprecedented attention and frequency of use from the community at this particular time due to its unique advantage of spanning distances. Patients can connect with doctors in different regions through the mobile medical platform and are no longer limited by geography. In addition, online access to medical care can effectively avoid contact infections caused by a large number of patients gathering in hospitals (1). In fact, with the development of modern Internet technology, mobile medical platforms have long been established, but their growth could be better. The main reason is that users need more trust in the mobile medical platform as they are worried about the effectiveness of online diagnosis and the leakage of their privacy, such as medical data (2–4). Therefore, improving users' trust in the mobile medical platform and attracting more users to use them is an effective way to alleviate the existing offline healthcare problems and deal with unexpected events (e.g., COVID-19).

Exciting research on users' use intention is mainly based on the framework of “trust-intention”(5). Regarding mobile medical platforms, some researchers have established the framework of “trust-intention” (6–8) but lacked a discussion on the effect mechanism. The mobile medical platform is more challenging to gain users' trust than other online platforms because they are directly related to users' health and life safety (9). It is essential to clarify the mechanism of users' trust in their use intention from the theoretical level. In addition, although the “trust-intention” framework has been generally accepted on the mobile medical platform, it still needs to introduce moderating variables to address the different contexts they may encounter (10). The relationship between trust and intention on the mobile medical platform is not static. Nevertheless, it can evolve dynamically depending on other conditions, so it is necessary to find more contextually appropriate variables and further investigate their moderating effects (6).

Scholars in the consumer field have extensively studied the use of intention and expanded the definition of intention (11). Dodds and William (12) proposed that consumers' purchase intention refers to the psychological intention before the purchase behavior occurs (12, 13). Some researchers pointed out that the intensity of purchase intention has an important impact on consumers' purchase behavior, or the generation of consumers' purchase behavior mainly depends on their purchase intention (14, 15). It has been argued that the theory of planned behavior model can help explain the rationale. The theory suggests that individual behavior is influenced not only by subjective norms and behavioral attitudes but also by some non-subjective factors (16). Based on this theory, scholars have further demonstrated a positive relationship between users' trust and their use intention, which is particularly significant in online shopping behavior (9, 17, 18). Scholars have attempted to construct a “trust-intention” framework for analyzing consumers' intention to purchase online. The “trust” refers to the users' trust in the platform, merchants, word of mouth, products, and other people and objects that the user may come into contact with during the online shopping process, which includes trust in brands, distributors, e-commerce platforms, other buyers, and products (19). The model can help researchers to explore the mechanisms by which different types of trust affect purchase intentions.

The intermediate mechanisms of the “trust-intention” framework have been studied in two ways, namely, perceived value and perceived risk. The studies of perceived value focus more on the balance between “profit” and “loss” (20). The perceived profit refers to the product's quality attributes, service experience, and technical support in a given situation that the users receive during the purchase and use (21). At the same time, the perceived loss is the total cost incurred by the user during the purchase process (22). Perceived risk is generated by user behavior and can be divided into two main aspects. One is uncertainty, which refers to the uncertainty of the performance and quality of the product itself (23, 24). The second is adverse consequences, which refer to the loss of time, money, and psychological damage caused by the purchase of products (24). These two aspects can also be summarized as objective and subjective risks. Perceived value and perceived risk provide an excellent theoretical basis for discussing this study's “trust-intention” mechanism. However, in this framework, we focus on the context in which these two mechanisms work and to what extent it requires consideration of the contextual factors, thus, the moderating variables need to be introduced for further analysis.

Based on the existing research, this study introduces two contextual variables, namely, personal innovation and technical risk concern to explore the contextual factors of the “trust-intention” framework. Personal innovation refers to an underlying personal characteristic of an individual's intention to accept new changes and ideas (25). Personal innovation is critical in the diffusion of new products as innovative users are the first to use when an innovative product is first introduced to the market (26). Existing research has already supported that personal innovation has an important impact on the use of mobile commerce (7). Technology risk concerns refer to users' concerns about privacy and security when using new technologies (27). Most existing studies classified privacy into information privacy, spatial privacy, and decision privacy, specifically as a personal home address, cell phone number, hobbies, physical characteristics, lifestyle habits, emotional experiences, etc., (28). In the internet context, sensitive personal information issues include being recorded and tracked when visiting websites, personally sensitive information being collected and used for product promotion or other purposes in unknown circumstances, personal information being sold to third parties without permission, and so on (29). Thus, privacy issues can affect users' intention to use the mobile Internet.

**Figure 1:**
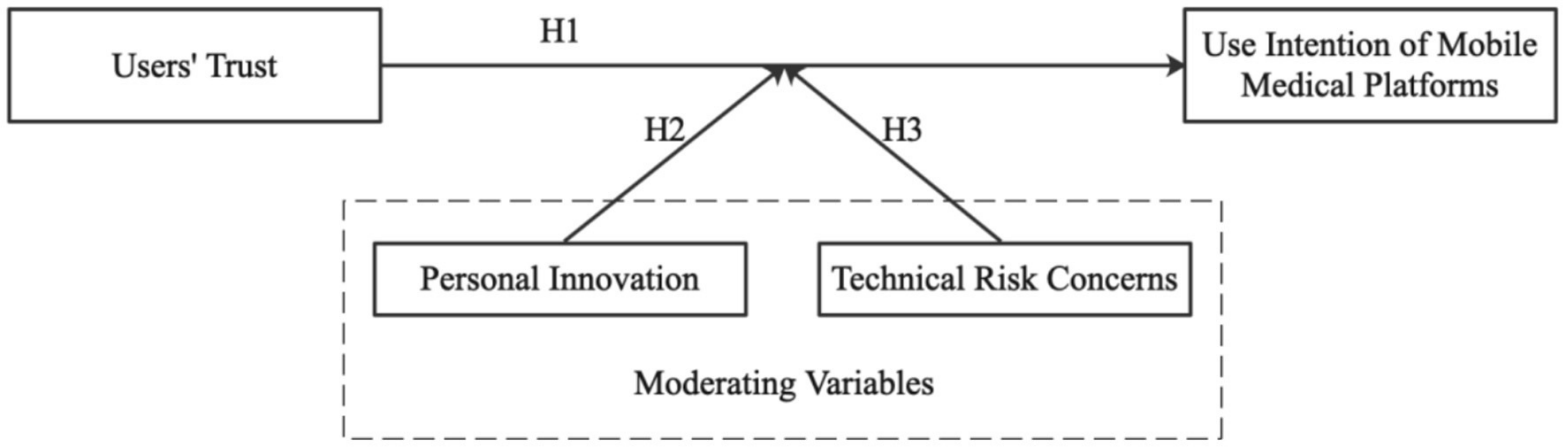
Conceptual framework of the research.

Based on the “trust-intention” framework, this study aims to elucidate the explanatory mechanism of “trust-intention” from the perspective of perceived value and perceived risk and introduces personal innovation and technical risk concerns as two moderating variables to further explore the framework in specific contexts. Therefore, this study establishes a theoretical framework, proposes corresponding research hypotheses, and empirically tests the hypotheses using a questionnaire survey. An overview of the conceptual framework of the study is shown in Figure 1.

## 2. Materials and methods

### 2.1. Hypotheses

#### 2.1.1. Users' trust in use intention of the mobile medical platform

Trust is critical in maintaining dependent exchange relationships and significantly impacts establishing and sustaining profitable long-term relationships (6). Users' trust can be considered an important emotional variable that supports the lasting relationship between products and consumers. Therefore, users' trust is crucial for the promotion of new products. The mechanism of the effect of users' trust on the use intention of the mobile medical platform in this study can be divided into two aspects.

First, amplify the perceived value. Perceived value is the users' overall evaluation of the effect of a product or service after weighing its perceived benefits against the costs paid (20). It combines the users' perceptual and rational perspectives (17, 18). The greater the users' trust in the mobile medical platform, the greater the perceived value. When online users trust the mobile medical platform, the mechanism of amplifying perceived value can achieve more profound dependence of users and transform this dependence and trust into a positive purchase and usage mindset (30), which further influences the use intention behavior (31) as reflected in the likelihood of recommending to others and the likelihood of repurchase behavior. Perceived value is a cognitive input that, once formed, has some persistence (32, 33). When users have a high level of trust in the platform, they will amplify its perceived value and, thus, be more willing to use the mobile medical platform.

Second, weaken the perceived risk. Perceived risk is that users will consider purchasing a product as rational economic agents and effectively avoid the risk during the purchase process (23). Prior research suggested that users' perceived risk reduced users' use intention (23, 34). Therefore, the seller must reduce the perceived risk to users. When online users trust the mobile medical platform, the weakened perceived risk mechanism can help companies reduce the perceived risk of users, which will enhance users' intention to purchase (35, 36). Furthermore, this trust allows users to ignore risks. Although such potential risks may pose future usage risks (37), ignoring risks can help maximize users' use intention for firms. When users have a high level of trust in the platform, they will weaken the perceived risk and, thus, be more willing to use the mobile medical platform.

The two mechanisms amplify the perceived value and weaken the perceived risk act on the relationship between trust and use intention. They have different temporalities, in which the amplifying perceived value tends to be more sustainable. In summary, this research proposes research Hypothesis H1:

Hypothesis 1 (H1): Users' trust positively affects their use intention of the mobile medical platform.

#### 2.1.2. Moderating impact of personal innovation

Personal innovation is an analysis of the individual characteristics of consumers, and it has been suggested that users who are early adopters of innovative technologies can be classified as innovators (38). Innovators tend to have a high level of personal innovation as they are more likely to make creative decisions and are less likely to be influenced by their experience (38). In addition, highly innovative people are always confident, more willing to experiment with new technologies, and more tolerant of new technologies (25, 39). Therefore, more innovative individuals are more likely to use the mobile medical platform.

First, personal innovation positively moderates the relationship between trust and use intention as groups with higher levels of personal innovation are more likely to search for positive news about new technologies and amplify their perceived value (3). Innovation diffusion studies have found that groups with higher levels of personal innovation have a higher intention to accept new technologies and are more likely to see positive reports when searching for them (22). These users gather information from different sources and develop positive technology endorsements, further enhancing and amplifying the perceived value of the mobile medical platform (7). Thus, the relationship between trust in the mobile medical platform and use intention will be further strengthened.

Second, personal innovation positively moderates the relationship between trust and use intention because groups with higher levels of individual innovation are optimistic about technological risks, thereby weakening perceived risks. Innovations are by nature uncertain, imprecise, and risky; the risks are potential and require users to perceive them. In innovation diffusion, groups with higher levels of individual innovation tend to be more tolerant of the risks of new technologies, thus, they are more willing to try them and are well prepared for the risks they may entail (7). Highly innovative groups are more focused on the potential benefits that technology may bring, thus weakening their concerns about risk (40). When using the mobile medical platform, they remain optimistic about the perceived possible risks of the platforms, which further weakens the perceived risks and, thus, strengthens the relationship between trust in the mobile medical platform and use intention.

Groups with higher levels of personal innovation tend to have positive attitudes toward technology and are more willing to accept the creation of a mobile medical platform. Thus, personal innovation tends to amplify the perceived value and weaken perceived risk, strengthening the relationship between trust in the mobile medical platform and use intention. In summary, this study proposes research Hypothesis H2.

Hypothesis 2 (H2): Personal innovation positively regulates the relationship between users' trust in the mobile medical platform and their use intention.

#### 2.1.3. Moderating impact of technical risk concerns

Technology risk concerns refer to users' concerns about the potential risks associated with new technologies (27). It has been argued that it is often detrimental to the adoption and diffusion of innovation when users have a high level of technical risk concern (41). Due to the concern about the risk of new technologies, users prefer to choose existing mature technologies and form path dependence. When users are highly reluctant to try new technologies, the innovation will be difficult to diffuse. Therefore, sellers need to minimize the technical risk concerns of users (42). For the mobile medical platform, high technology risk concerns mean that users are concerned about privacy information leakage (28). This group also tends to have a negative attitude toward technology and is reluctant to accept the mobile medical platform as a new format.

First, groups with high levels of technical risk concerns tend to be pessimistic and reluctant to amplify perceived value, which will negatively moderate the relationship between trust and use intention. Groups with high levels of technical risk concern tend to be pessimistic about adopting new technologies and believe that their use may pose technical risks (30). The services provided by the mobile medical platform are emerging healthcare services still in the exploratory stage. People need to learn more about mobile medical service providers' operations and related systems and may perceive high privacy risks associated with uploading personal medical data on the Internet (43). In particular, third-party platforms are often private companies rather than public hospitals, making security challenging to guarantee (41). As a result, they are reluctant to trust the platform. They need to be aware of the value that the mobile medical platform may bring to healthcare services and are reluctant to explore new technological innovations (42). Thus, the high-tech groups weaken the relationship between users' trust in the mobile medical platform and their use intention by not amplifying or even reducing the perceived value.

Second, groups with higher technical risk concerns have fearful attitudes toward technology, enhancing perceived risk and negatively moderating the relationship between trust and use intention. Groups with high levels of technical risk concerns tend to fear new technologies, seeing them as “poison” that disrupts their lives and equating technology with risk (44). Groups with higher levels of technical risk concerns may even develop a fear of new technologies. The increase in perceived risk tends to weaken the relationship between users' trust in the mobile medical platform and their use intention. Internet platforms' existing privacy and security issues further reinforce users' perceptions of the risks of the mobile medical platform (45). People believe that Internet platform companies are bound to have privacy issues, such as information leakage from social networks (4). As a result, they are reluctant to trust platforms, and their risk perceptions of platforms are enhanced.

Groups with higher levels of technical risk concerns are pessimistic or even fearful of technology and are reluctant to accept the creation of the mobile medical platform. In this process, they tend to amplify the perceived value but strengthen the perceived risk, weakening the relationship between users' trust in the mobile medical platform and their use intention. In summary, this study proposes the research Hypothesis H3.

Hypothesis 3 (H3): Technical risk concerns negatively affect the relationship between users' trust in the mobile medical platform and their use intention.

### 2.2. Data collection

The sample for this study was the mobile medical platform users. The survey period was from February to May 2020 during COVID-19, in which the online diagnosis for individuals could be more convenient due to the need for urban spatial isolation. What is more, our research subjects are users of mobile medical platforms, who are more familiar with online research methods. We can obtain valid data by this way. This is because treatment at offline hospitals increases the risk of infection, and hospitals are primarily responding to the epidemic. In addition, many cities and regions have adopted quarantine measures such as suspending public transportation, making offline access to medical care more difficult. In this context, exploring the intention of using the mobile medical platform makes more sense. This study commissioned Wenjuanxing Inc. (WJX), a professional market research firm, to conduct this survey. The researchers randomly sampled from the WJX sample database and sent the questionnaire *via* email from 1 February to 1 May 2020. The questionnaire was filled out anonymously to ensure the authenticity of the answers, and the researchers returned a total of 678 questionnaires. Based on this, the study eliminated the sample according to the following principles: (1) the presence of missing answers and (2) the apparent regularity of the answers. Finally, the study obtained a total of 625 valid questionnaires.

### 2.3. Statistical analysis

#### 2.3.1. Instrumentation

According to the research variables, three instruments were used to collect the required data. Through extensive literature reading and research, the researcher integrated and adapted these three research instruments to fit the situation and the main research objectives of this study. The questionnaire was divided into four sections, including control variables, independent variables, dependent variables, and moderating variables. Table 1 summarizes the details of each section.

**Table 1:** The detailed information in each section.

**Type**	**Variable**	**Items**	**Source**
Control variables	Age	Age	
	Gender	Gender	
	Education	Education	
	Epidemic regions	In the high-risk regions or not	
Independent variable	Users' trust	TR1 I believe the platform has the expertise to ensure the safety of the service.	McKnight et al. (44)
		TR2 I believe that the platform service providers provide medical services with good quality.	
		TR3 I believe that the content of the platform is accurate.	
		TR4 The platform provider will follow its promises to meet its obligations.	
		TR5 If I need it, the platform will help as much as possible.	
		TR6 I trust that the platform service provider will not undermine my interests for its own sake.	
		TR7 I trust that I can perform medical services on the platform.	
Dependent variable	Use intention on the mobile medical platform	UI 1 I plan to use the mobile medical platform services in the next 3 months.	Johnston and Warkentin (46)
		UI 2 I predict that I will use the mobile medical platform services within the next 3 months.	
		UI 3 I plan to use the mobile medical platform services in the next 3 months.	
Moderating variable	Personal innovation	PI 1 I am willing to adopt new information technology.	Lee (10)
		PI 2 I think it is very interesting to try new information technology.	
		PI 3 I like trying new information technologies.	
	Technical risk concerns	TRC1 My use of mobile medical services would result in my loss of control over the privacy of my personal health information.	Cocosila and Archer (47)
		TRC2 Using mobile medical services would result in my loss of privacy because my personal health information could be used without my knowledge.	
		TRC3 If I use mobile medical services, other people may have control over my health information.	

The independent, dependent, and moderating variables are measured using a 7-point Likert scale, where 1 indicates strong disagreement and 7 indicates strong agreement. The control variables include age, gender, educational background, and epidemic regions (the Chinese government made judgments based on the number of cases in the COVID-19 epidemic regions and divided the region into high-risk regions and low-risk regions). Since this study was conducted in the specific context of COVID-19, the degree of variation in epidemic regions affects users' trust in healthcare, thus, the epidemic regions are included as control variables in this study.

#### 2.3.2. Validity and reliability

In the reliability test, Cronbach'α coefficient of trust is 0.92 (>0.7), indicating that the internal structure of the trust scale has a good fit. The trust scale in this study is adapted using mature scales in appropriate contexts and has good content validity. The result of the factor analysis is shown in Table 2, and it can be summarized that all variables have passed the sphericity test. The factor loading value of each item is >0.6, which indicates good aggregation validity. Based on this, it can be concluded that the reliability and validity of the scales are good and can be used for further regression analysis and discussion.

**Table 2:** Factor analysis results (validity test).

**Item 1**	**Factor loading**	**Item 2**	**Factor loading**	**Item 3**	**Factor loading**	**Item 4**	**Factor loading**
Trust (1)	0.936	Intention (1)	0.960	PI (1)	0.940	TRC (1)	0.930
Trust (2)	0.906	Intention (2)	0.844	PI (2)	0.854	TRC (2)	0.857
Trust (3)	0.903	Intention (3)	0.895	PI (3)	0.885	TRC (3)	0.823
Trust (4)	0.875	Sphericity test	Significant (0.000)	Sphericity Test	Significant (0.000)	Sphericity test	Significant (0.000)
Trust (5)	0.940						
Trust (6)	0.880						
Trust (7)	0.910						
Sphericity test	Significant (0.000)						

PI, personal innovation; TRC, technical risk concerns.

#### 2.3.3. Regression test

##### 2.3.3.1. Multicollinearity test

Existing studies have used correlation coefficients between variables and variance inflation factors (VIFs) to test whether multiple linear regression models have multicollinearity problems. According to the results of the VIF test, when the VIF is >10, there is a multicollinearity problem among variables. The test results of this study showed that the VIF of all variables did not exceed 10, and the corresponding tolerances were all >0.1, indicating no multicollinearity problem.

##### 2.3.3.2. Deviation of the same source method

According to the conclusions of previous studies (48), the results of this study were analyzed using Harman's single-factor test. The results met the criteria, and the measurement bias did not affect the study's conclusion.

The empirical tests of this article are in the following order: first, descriptive statistics are done according to the sample situation. Second, the Pearson correlation test is done to test the correlation between the variables according to the regression analysis method. Finally, the OLS least square method is used for the regression test. All of the aforementioned processes are performed using STATA statistical software, and the specific results are shown in the result section.

## 3. Results

### 3.1. Descriptive statistics

The samples' demographic information is shown in Table 3, which mainly shows the following characteristics: (1) Age: The data show that the proportion of users in the two categories of 21–30 years old and under 20 years old is high. People in these age groups are more receptive to new things and willing to use the mobile medical platform and social networks. (2) Gender: men accounted for 62.4%, and women accounted for 37.6%. (3) Educational background: The sample population is mainly concentrated in universities and postgraduates, and these groups use mobile social networking more frequently. (4) Epidemic region: The proportion from high-risk regions was 50.4%, and low-risk regions accounted for 49.6%.

**Table 3:** Basic information about the participants.

**Demographic information**	**Items**	**Number**	**Percentage (%)**
Age	<20	183	29.28
	20–30	367	58.72
	31–40	49	7.84
	41–50	23	3.68
	>50	3	0.48
Gender	Men	390	62.4
	Women	235	37.6
Educational background	Below University	2	0.32
	Undergraduate	441	70.56
	Postgraduate	182	29.12
Epidemic regions	High-risk regions	315	50.4
	Low-risk regions	310	49.6

### 3.2. Correlation statistics

The results of the Pearson correlation coefficients between the variables are listed in Table 4. Pearson correlation analysis reflects the correlation between variables and provides references for the rationality of the model and assumptions.

**Table 4:** Results of Pearson correlation coefficients.

	**Age**	**Gender**	**Education**	**Area**	**Trust**	**UI**	**PI**	**TRC**
Age	1							
Sex	0.0661^*^	1						
Education	0.1976^***^	0.3084^***^	1					
Area	0.0444	0.0227	−0.1375^***^	1				
Trust	0.0239	0.0247	0.0249	−0.0182	1			
UI	0.0129	−0.0001	0.0189^*^	−0.0184	0.8525^***^	1		
PI	0.0242	−0.0069	0.0140	0.0194	0.4975^***^	0.7898^***^	1	
TRC	−0.0265	−0.0262	−0.0202	0.0183	0.0910^**^	−0.1603^***^	−0.3390^***^	1

UI, use intention; PI, personal innovation; TRC, technical risk concerns.

*** p < 0.01,

** p < 0.05,

* p < 0.1.

### 3.3. Regression analysis results

The regression test results are shown in Table 5.

**Table 5:** Regression analysis results.

	**Model 1 (Direct effect)**	**Model 2 (Indirect effect)**	**Model 3 (Indirect effect)**
Age	−0.0183(0.0546)	−0.0365(0.0320)	−0.0408(0.0460)
Sex	−0.9259(0.0914)	−0.0437(0.0535)	−0.1065(0.0770)
Education	0.0249^*^(0.0965)	0.0218^*^(0.0567)	0.0354(0.0813)
Area	−0.0049^*^(0.0852)	−0.0574(0.0499)	0.0271(0.0718)
Trust	0.8777^***^(0.0216)	0.5454^***^(0.0276)	1.0783^***^(0.0279)
Personal Innovation		0.3558^***^(0.0289)	
Trust × personal innovation		0.0215^***^(0.0061)	
Technical risk concerns			0.0581^*^(0.0326)
Trust × technical risk concerns			−0.0621^***^(0.0073)
Constant term	0.9536^***^(0.3301)	0.3164(0.2079)	0.7628^**^(0.2942)
R^2^	0.7272	0.9068	0.8069
Adjust R^2^	0.7250	0.9058	0.8047
Number	625	625	625

*** p < 0.01,

** p < 0.05,

* p < 0.1.

#### 3.3.1. Direct effect test

This study empirically tested the relationship between users' trust in the mobile medical platform and their use intention. The dependent variable of Model 1 is use intention, and this model contains control variables and independent variables (trust). From Model 1, the data showed that trust positively affects the use intention. The coefficient of the path was 0.8777, and a P-value of < 0.01, which means that trust improves users' use intention. Thus, H1 is supported.

#### 3.3.2. Indirect effect test

Based on the H1, this study introduces two moderating variables: personal innovation and technical risk concerns. The empirical results are shown in Models 2 and 3 in Table 5. The dependent variable is both the use intention. Model 2 contains control variables, independent variables (trust), moderating variables (personal innovation), and cross terms (trust × personal innovation), while Model 3 contains control variables, independent variables (trust), moderating variables (technical risk concerns), and cross terms (trust × technical risk concerns). Hypothesis 2 states that personal innovation moderates the relationship between trust and use intention. According to Model 2, the moderating effect is significant (r = 0.0215, P < 0.01), and H2 is supported. Similarly, the moderating effect of technical risk concerns is also significant (r = −0.0621, P < 0.01), supporting the H3.

## 4. Discussion

This article focuses on developing a mobile medical platform in the field of public health during COVID-19. Through the survey results of 625 individuals, it is found that there is a positive correlation between trust and the use of the mobile medical platform. The relationship between trust and use intention is explained through two interpretation mechanisms, namely, perceived value and perceived risk. On this basis, two moderating variables of personal innovation and technical risk concerns are introduced. The empirical results show that personal innovation positively moderates the relationship between trust and use intention. At the same time, technical risk concerns have a negative impact on the relationship between trust and use intention. This provides new inspiration for the development of the mobile medical platform.

### 4.1. Theoretical contribution

The research conclusions further promote the development of a mobile medical platform for public health, especially during COVID-19 (49). This research discussed the users' intention to accept and use the mobile medical platform starting from the theoretical background of new technology promotion and users' use intention.

The theoretical innovation of this research is mainly reflected in two aspects. One is to extend the academic research of use intention to the specific context of the mobile medical platform. Traditional research on use intention is mainly based on the consumer domain, such as e-commerce platforms (12, 14, 15, 23). As an online channel for medical services, the mobile medical platform differs from the general consumption field. Medical services are activities that are closely related to people and their life and health. If there is a problem with purchasing goods online, it can be achieved by returning or exchanging them. However, this logic only holds on the mobile Internet platform, where accidents are often fatal and irreversible (23, 24, 34). Users are reluctant to engage in “trial and error” with healthcare services. Therefore, users' trust is more critical on mobile Internet platforms, and it is not easy to achieve sustainable development without users' trust. Users' trust in the mobile medical platform is the key to developing the mobile medical platform (6, 8). This study provides a new dimension to the field of mHealth-related research. Second, it enriches the research framework of “trust-intention”. Based on the basic framework of “trust-intention”, this study introduces two mechanisms, namely, perceived value and perceived risk. On this basis, the researchers introduce personal innovation from individual characteristics and technical risk concerns from the mobile medical platform, further enriching the basic framework of “trust-intention”.

The positive relationship between trust and use intention in the mobile medical platform is consistent with the findings of previous research on “trust-intention” that trust promotes use intention (6, 8). This study is more meaningful in exploring the relationship between trust and uses intention in the context of COVID-19. Perceived value and perceived risk are based on trust, where perceived value enhances trust, and perceived risk weakens trust. In this research, the researchers identify the relationship between trust and intention by starting from the two mechanisms mentioned earlier. The results of this study are a reaffirmation of the “trust-intention” framework.

H2 illustrates the positive moderating impact of personal innovation on the relationship between trust and use intention. Similar to the theory of new product adoption, this research believed that personal innovation is essential in expressing their use intention. When individuals are more receptive to innovative affairs, they are more willing to try emerging things such as mobile medicine (7, 26, 50). Furthermore, this research is also a new application of new products in the medical field.

The result from the testing of H3 predicts that technical risk concerns moderate the relationship between trust and use intention. Internet technology has two sides. On the one hand, it improves users' convenience, such as mobile medical and e-commerce. On the other hand, it also brings risk challenges to daily life, such as data leakage. For this reason, technical risk concerns must be considered in the research of the mobile medical platform. This study takes technological risk concerns as a moderating variable and empirically tests that technological risk concerns negatively moderate the relationship between trust and use intention.

### 4.2. Management implication

The current study provides several managerial implications for the practice of public administration in the context of public health.

First, promoting the mobile medical platform to address public health and medical issues requires building users' trust. The development of the mobile medical platform is an effective complement to the existing healthcare system as the development and improvement of the mobile medical platform can help expand the scope of healthcare services and improve efficiency. However, the development of the mobile medical platform is not satisfactory at present because the trust of users has not been established. Therefore, the findings of this study provide a practical reference for building users' trust in practice. The study concluded that platforms need to enhance users' perceived value and reduce their perceived risk to improve their trust in the platform and, thus, their willingness to use it. COVID-19 was a critical period for the development of the mobile medical platform (49, 50). For safety reasons, more and more users began to try to address relatively minor medical problems through mobile healthcare at home. At this time, it is essential to make users perceive the value of the mobile medical platform.

Second, it is vital for individuals to actively cultivate the spirit of innovation and learn to accept new things. Personal innovation positively impacts the “trust-intention” framework, and the more innovative a group is, the more willing it is to use the mobile medical platform. This means that different groups of individual innovators have other intentions to use the mobile medical platform (3). Therefore, there is a need to develop personal innovation skills and increase the acceptance of new technologies by individuals. Developing new technologies inevitably involves certain risks, but accepting new technologies contributes to their effective development. For example, online shopping was questioned in its early stage, but many problems were solved by establishing policies and technological improvements after decades of development. Similarly, the development of the mobile medical platform requires greater space and opportunity from individuals.

Third, the mobile medical platform should avoid technical risks and reduce the perceived risks to users. For the mobile medical platform, it is critical to reduce the perceived risk to users, especially the privacy issues that come with technology. Therefore, platforms need to pay great attention to the adverse effects of technology and reduce users' perceived risk through various methods. First, it can be updated through technology to solve the problem of privacy leakage brought about by insufficient technology. Second, it can introduce internal rules of the platform to avoid privacy leakage through standard norms and conventions. Third, it can increase publicity. Information asymmetry is the main reason for users' distrust of the platform. Promotion can reduce information asymmetry and make more users participate in using the mobile medical platform.

Fourth, the government should recognize the importance of the mobile medical platform to public health development and guide users to adopt the mobile medical platform through policies. As a policymaker, the government should recognize that the mobile medical platform can effectively alleviate the problems of difficult access and complicated processes and help more people break through the limitations of distance. For this reason, the development of the mobile medical platform has a significant role in improving public health services. Especially in the COVID-19 period, when medical resources are scarce, the mobile medical platform can realize the efficient allocation of medical help. For the government to effectively guide more users to use the mobile medical platform through policies to reduce the pressure of offline medical care, it is necessary to start from the policy aspect to guide the benign development of users, the mobile medical platform, and medical service providers.

### 4.3. Limitation and future research

Despite the contributions of this study, there are limitations, some of which can be the basis for future research. First, this research used the survey method to verify the research hypotheses. Using experimental methods can improve the external validity of conclusions, especially in the context of COVID-19. The moderating effect of personal innovation and technical risk concerns can be further verified through experiments. Second, the data sources of this study are mainly from China. The perception of internet healthcare in China is different from that of other regions, thus, in other countries or regions, the external validity of the conclusions will all be reduced. Future research can test the findings of this article in different countries.

## Data availability statement

The original contributions presented in the study are included in the article/supplementary material, further inquiries can be directed to the corresponding author.

## Ethics statement

Ethical review and approval was not required for the study of human participants in accordance with the local legislation and institutional requirements. Written informed consent from the patients/participants was not required to participate in this study in accordance with the national legislation and the institutional requirements.

## Author contributions

The author confirms being the sole contributor of this work and has approved it for publication.
